# A Rare Case of Junctional Bradycardia Secondary to Oral Phenytoin

**DOI:** 10.7759/cureus.25251

**Published:** 2022-05-23

**Authors:** Bilal A Niazi, Chinmay Trivedi, Benjamin Perrella

**Affiliations:** 1 Internal Medicine, Hackensack Meridian Health - Palisades Medical Center, North Bergen, USA

**Keywords:** sodium channel, toxicity, hypotension, junctional bradycardia, phenytoin

## Abstract

Phenytoin is a commonly used anti-seizure agent, which stabilizes neuronal membranes by blocking voltage-gated sodium channels to inhibit the propagation of action potentials during convulsions. However, phenytoin has also been shown to have antiarrhythmic effects as it can prolong the effective refractory period of ventricular pacemaker cells. Adverse cardiac effects such as junctional bradycardia are usually seen with intravenous use. Cardiovascular dysfunction is not well recognized in oral phenytoin toxicity. Here we present a case of junctional bradycardia due to oral phenytoin toxicity, which resolved spontaneously with the discontinuation of phenytoin. This case report will serve to increase awareness of the adverse cardiovascular effects of oral phenytoin toxicity to improve the recognition and treatment of these adverse effects.

## Introduction

Phenytoin is a drug commonly used to treat epilepsy that inhibits sodium channels to prevent neuronal action potentials and decrease the spread of seizures [1]. Phenytoin is known to have the adverse effects of gingival hyperplasia, lupus-like hypersensitivity, encephalopathy, sedation, and hyperkinesia [2]. However, adverse cardiac reactions such as arrhythmias are less commonly recognized and usually seen in intravenous phenytoin use, dependent on loading dose and infusion rate [3]. Phenytoin has class 1b antiarrhythmic properties and has been used to suppress arrhythmias such as ventricular tachycardia [4]. There is paucity in the literature on the cardiotoxic effects of phenytoin when given orally. Here we present a case of a patient taking oral phenytoin who was found to have junctional bradycardia with subsequent hypotension in the setting of elevated phenytoin levels. This case report will bring awareness of the cardiotoxic effects of oral phenytoin to aid in the expedient recognition and treatment of potentially fatal cardiotoxicity.

## Case presentation

An 82-year-old female with a past medical history of epilepsy and intellectual disability presented to the Emergency Department (ED) for hypotension and lethargy from a nursing home. A review of systems was not possible due to the patient's intellectual disability, however, her past medical history and medications revealed that she was taking 100 mg phenytoin tablets every 8 hours and 40 mg atorvastatin tablets daily. No recent medication changes were noted in the review of medical history. On arrival, her vital signs revealed a temperature of 97.6 F, a heart rate of 38, blood pressure of 87/53 mm Hg, respiratory rate of 16 breath cycles/min, and oxygen saturation was 100% on room air. Her physical exam was unremarkable for trauma, abnormal extraocular movements or pupillary response, wheezing or rhonchi on lung auscultation, elevation in JVD, or lower extremity swelling.

**Figure 1:**
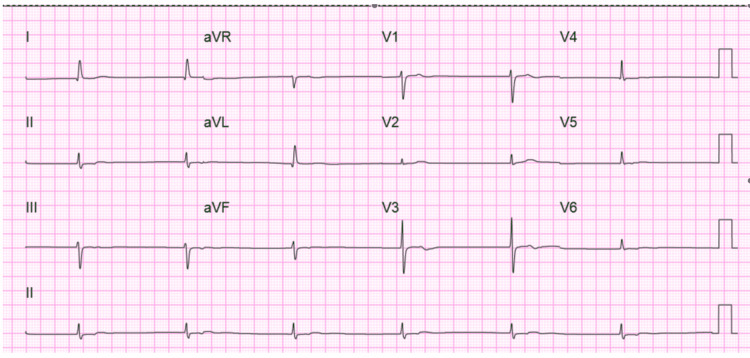
12-lead electrocardiogram on presentation demonstrating junctional bradycardia with heart rate < 40 beats  per minute.

Her laboratory evaluation was remarkable for elevated total phenytoin levels at 44.1 mcg/mL (therapeutic range: 10-20 mcg/mL). She did not have any previous phenytoin levels measured. Her albumin level was 4.2 g/dL, troponins were < 0.02 ng/dL, and serology for Chagas disease and Lyme disease were undetectable. Her laboratory evaluation was otherwise within normal limits for WBC, Hgb, electrolytes, TSH, procalcitonin, and lactic acid. EKG on arrival revealed a junctional rhythm with a heart rate of 36, as shown in Figure 1.

Chest x-ray was unremarkable for any consolidation, congestion, effusion, or pneumothorax. Two liters of normal saline were administered with an improvement of her blood pressure to 124/67. Phenytoin was discontinued and cardiac monitoring was initiated. Transcutaneous pacing pads were made available at the bedside. Her phenytoin levels progressively trended down per daily assessment as shown in Table 1.

**Table 1:** Serum levels of phenytoin The progressive downtrend of serum phenytoin levels after being discontinued.

	Hospital Day 1	Hospital Day 2	Hospital Day 3	Hospital Day 4
Phenytoin level (microgram/mL)	44.1	33.3	28.3	18.7

EKG on the following day revealed spontaneous conversation to sinus rhythm with a heart rate of 53 beats per minute. Cardiac monitoring through her five-day hospital course did not reveal any abnormal rhythms. Intravenous levetiracetam was started on hospital day 4 and transitioned to oral levetiracetam on hospital day 5. She remained seizure-free throughout her entire hospital course. A transthoracic echocardiogram was performed which revealed an ejection fraction of 60%-65% and otherwise did not reveal any structural abnormalities. Her blood pressure remained in the systolic range 120s and diastolic range 80-90s through the remainder of her hospital stay and no fevers or abnormal changes in her WBC were noted. Ultimately, her bradycardia was attributed to oral phenytoin toxicity and she was discharged on oral levetiracetam to manage her epilepsy.

## Discussion

Phenytoin is an anti-epileptic medication that can be administered intravenously or orally. The anti-epileptic effects are likely achieved by inhibitory effects of phenytoin on voltage-gated sodium channels on neurons, which reduces convulsion activity [1]. However, phenytoin has also been shown to have cardiac effects and is classified as a class 1b antiarrhythmic [4]. The most common presenting symptoms of phenytoin toxicity include unsteady gait, weakness, dizziness, drowsiness, and nausea/vomiting [5]. Adverse cardiac effects of phenytoin toxicity include bradycardia, hypotension, and arrhythmias [6]. However, these effects are usually seen with intravenous use, dependent on the rate of administration [7]. It is therefore currently recommended to initiate cardiac monitoring during the administration of intravenous phenytoin [8]. Oral administration of phenytoin is not well recognized to cause adverse cardiac effects and documented rarely.

Our patient taking oral phenytoin was found to have junctional bradycardia and elevated phenytoin levels. A junctional rhythm is described as a cardiac rhythm originating from the atrioventricular node or bundle of His as opposed to the sinoatrial node [9]. Causes of junctional bradycardia include ischemia of the atrioventricular node, electrolyte abnormalities, drug toxicities, myocarditis, and infections like Chagas disease and Lyme disease [10]. Our patient’s troponin levels were within normal range and there were no characteristics of ischemia on EKG. Her electrolytes were unremarkable. She was afebrile without abnormal changes to her white blood count to suggest infection. Serum serology for Chagas and Lyme disease was unremarkable. After ruling out the well-known causes of junctional bradycardia as described earlier, oral phenytoin was found to be the likely cause of junctional bradycardia, considering the elevated phenytoin levels.

Phenytoin has a narrow therapeutic window and wide inter-individual metabolic variability [10]. Oral phenytoin is absorbed in the gut and metabolized in the liver. The most common causes of elevated phenytoin levels include excessive self-medication, misunderstanding prescription orders, drug interactions, and unknown causes [10]. Drugs such as folic acid, dexamethasone, and rifampicin have been known to increase phenytoin serum levels [11]. Our patient presented from a nursing home where medication administration was regulated and she was not taking any medications known to interact with phenytoin levels.

Phenytoin variably works on sodium channels on cardiac myocytes leading to dysfunction at the site of the atria and atrioventricular (AV) node. Phenytoin can effectively enhance or delay the refractory period leading to Tachy-Brady syndromes [11]. Interestingly, phenytoin does not appear to affect the His-Purkinje bundle [12]. Phenytoin does not have an effective antidote and treatment is discontinuing phenytoin and supportive management of symptoms [13]. Bedside pacing and atropine should be readily available for bradycardia, and intravenous fluid management and vasopressors should be considered for resistant hypotension. Our patient’s junctional arrhythmia converted spontaneously to sinus rhythm after discontinuing phenytoin without requiring atropine or cardiac pacing.

## Conclusions

This case report highlights the potential adverse cardiovascular effects of oral phenytoin toxicity. Bradycardia and hypotension can be seen with intravenous administration of phenytoin but are not well recognized with oral administration. Phenytoin is a commonly used anti-epileptic medication, which inhibits voltage-gated sodium channels on neurons to limit the spread of convulsions. However, it is known to have effects on cardiac cells and has even been used as an antiarrhythmic. Here we present a case of junctional bradycardia in the setting of oral phenytoin toxicity, which resolved spontaneously with discontinuation of phenytoin. This case report will serve to bring awareness of the potential adverse cardiac effects of oral phenytoin ingestion as they are not a well-recognized complication.
